# The extent of using mobility assistive devices can partly explain fatigue among persons with late effects of polio – a retrospective registry study in Sweden

**DOI:** 10.1186/s12883-016-0753-6

**Published:** 2016-11-18

**Authors:** I. Santos Tavares Silva, K. S. Sunnerhagen, C. Willén, I. Ottenvall Hammar

**Affiliations:** 1 Department of Health and Rehabilitation, Institute of Neuroscience and Physiology, The Sahlgrenska Academy, University of Gothenburg, Gothenburg, Sweden; 2 Department of Occupational Therapy and Physiotherapy, The Sahlgrenska University Hospital, Gothenburg, Sweden; 3 Department of Clinical Neuroscience and Rehabilitation, Institute of Neuroscience and Physiology, University of Gothenburg, Gothenburg, Sweden; 4 Sunnaas Rehabilitation Hospital, Nesodden, Oslo Norway; 5 Centre of Aging and Health-AGECAP, University of Gothenburg, Gothenburg, Sweden

**Keywords:** Post poliomyelitis syndrome, Occupational therapy, Multidimensional fatigue inventory (MFI-20) scale, Cross-sectional studies

## Abstract

**Background:**

Fatigue is reported as one of the most disabling symptoms and is common among persons living with late effects of polio. Although fatigue has been studied in the context of people living with late effects of polio, there is a lack of knowledge concerning the association of fatigue and variables of importance for participation in daily life. Therefore, the aim of this study was to explore possible factors associated with fatigue among persons with late effects of polio in Sweden.

**Methods:**

This retrospective registry study consisted of 89 persons with late effects of polio living in Sweden. Fatigue was measured with the Multidimensional Fatigue Inventory (MFI-20) scale, Swedish version. Pearson’s correlation coefficient was used to analyse the correlation between the factors and fatigue, and a multiple linear regression was carried out to explore factors for fatigue.

**Results:**

Fatigue statistically significantly correlated with age (*r* = 0.234, *p* < 0.05) and the use of mobility assistive devices (*r* = 0.255, *p* < 0.05). The multiple linear regression model showed that the factors age (β = 0.304, *p* < 0.019) and mobility assistive devices (β = 0.262, *p* < 0.017) were associated with fatigue among persons living with late effects of polio, and the model partly explained 14% of the variation of fatigue.

**Conclusions:**

Fatigue could partly be explained by the extent of using mobility assistive devices and age. Healthcare professionals should provide and demonstrate the importance of assistive devices to ensure management of fatigue in persons living with late effects of polio.

## Background

Fatigue is a common symptom reported among persons living with late effects of polio [1–3]. There are ambiguously definitions of fatigue in the literature [4–7]. It can be assessed in different ways [5], and is seen as a complex phenomenon with a large number of conceptualizations [6]. Fatigue can be defined as an overwhelming sense of tiredness, a lack of energy, and feeling of exhaustion, that can be associated with impaired physical and/or cognitive function [6]. In this study, fatigue is defined as an overwhelming sense of tiredness, a lack of energy and a feeling of exhaustion that may be associated with the impaired physical and/or cognitive function [6]. Fatigue may be either peripheral or central depending on where in the nervous system it originates [8], and there are several types of fatigue, such as general, physical or muscle fatigue [3].

About 60–85% of persons living with late effects of polio face recurrence of the original polio related manifestations as well as new symptoms, suggesting new health problems [9]. This state is usually named post-polio syndrome (PPS). People may experience late effects of polio without being diagnosed with PPS [3]. Several terms have been used to describe the symptoms followed by polio, such as ‘the late effects of polio’ and ‘post-polio sequelae’ [10]. In this study the term ‘the late effects of polio’ is used. According to Gawne and Halstead [10], the specific criteria for PPS are: a prior episode of paralysis confirmed by history, physical exam, and findings on an electromyogram (EMG); a period of neurological recovery followed by an extended interval of neurological and functional stability, usually lasting 20 years or more; and a gradual or abrupt onset of new neurogenic, non-disuse weakness in previously affected and/or unaffected muscles. These symptoms may or may not be accompanied by other new health problems, such as excessive fatigue, muscle pain, joint pain, decreased endurance, decreased function, and atrophy. Other neurologic, medical and orthopaedic conditions that might cause the health problems mentioned above must be excluded [10].

Persons living with late effects of polio may face restrictions on participating in different areas of life [11, 12]. Restrictions have been identified in occupations related to their family role, work and education, and to autonomy outdoors [11]. Dependence in occupations such as cleaning, transportation, and shopping has also been associated with restrictions in daily life activities [12]. Fatigue has been pointed out as an invisible disabling symptom affecting a person’s ability to perform occupations and limiting mastery of daily occupations [13]. It has also been reported that persons living with late effects of polio act at close to maximum of their capacity, and fatigue has been found to be related to muscle overuse [14, 15]. The perceived fatigue can remain for days after fatiguing exercise but without reduction. This phenomenon called low frequency fatigue can be described as persisting for prolonged periods of time before full capacity has returned [16, 17].

Earlier research [11–13, 18, 19] has identified various factors as important when exploring fatigue in everyday life among persons living with late effects of polio. The explanatory power of these factors in relation to fatigue has not yet been captured. In this study we explored fatigue in relation to the factors age, gender, country of birth, occupational/employment level and, the use of mobility assistive devices. Research has shown that there are inequities in health status associated with country of birth [18, 19]. The level of occupation/employment has been shown to be associated with fatigue [20]. Studies [21, 22] have shown that persons living with late effects of polio experience tiredness which restricts participation at work. These limitations resulted in both physical and psychosocial distress created by an internal conflict between what the person want to do and what they are able to do [21, 22]. Moreover, another factor of importance for fatigue is the use of assistive devices, which has shown to positively affect ability and reduce perceived difficulties [12]. Research [23] has shown that persons living with late effects of polio could experience pain and fatigue when they hesitated to start using assistive devices, in particularly mobility devices. To get a better understanding of factors associated with fatigue, the objective was to explore possible factors associated with fatigue among persons with late effects of polio in Sweden.

## Methods

### Sampling and participants

This retrospective registry study used patient data from a clinical database with persons with late effects of polio from the Polio Clinic, Sahlgrenska University Hospital, Gothenburg, Sweden. The clinic has encountered more than 900 persons with polio since the clinic opened 1994, and since that time different questionnaires have been used [24]. In the present study, data from persons that responded to a questionnaire concerning fatigue were identified from the clinical database. The following inclusion criteria were used: *(i)* persons aged 18 years or older, and *(ii)* with late effects of polio. In a total, 89 persons were included in the study; those who had answered the fatigue questionnaire. Due to internal dropouts on different items the calculations were based on slightly different sample size (see notes in Tables 2, 3, 4, and 5).

### Assessments

Data were collected between 2008 and 2011. The dependent variable of fatigue was explored and analysed in relation to the following factors; gender, age, country of birth, occupation/employment level, and the use of mobility assistive devices.

#### Dependent variable

Fatigue was assessed using the Swedish version of the Multidimensional Fatigue Inventory (MFI-20) [5], which is a self-administered questionnaire with a total of 20 items. The MFI-20 assesses a person’s self-rated fatigue in the following five subscales: General Fatigue (GF), Physical Fatigue (PF), Mental Fatigue (MF), Reduced Motivation (RM), and Reduced Activity (RA). The subscales and the 20 items are presented in Table 1. The level of fatigue can be scored with a minimum of 4 points to a maximum of 20 points on the 5-point Likert scale (1 = yes, that is true, 2–4 = no verbal specification, and 5 = no, that is not true). A total fatigue score ranging from 20 to 100 can be calculated as the sum of the five subscales scores. A higher score represents higher levels of fatigue. The Swedish version of the MFI-20 has been psychometrically tested, showing good psychometrically properties in different settings (persons with post-polio, cancer, fibromyalgia, chronic widespread pain and in a healthy population) [5, 25, 26].

**Table 1:** Subscales and items within the MFI-20

MFI-20 Subscales	Item nr	Item
General fatigue	1	I feel fit
	5	I feel tired
	12	I am rested
	16	I tire easily
Physical fatigue	2	Physically I feel only able to do a little
	8	Physically I can take on a lot
	14	Physically I feel I am in a bad condition
	20	Physically I feel I am in an excellent condition
Mental fatigue	7	When I am doing something, I can keep my thought on it
	11	I can concentrate well
	13	It takes a lot of effort to concentrate on things
	19	My thoughts easily wander
Reduced activity	3	I feel very active
	6	I think I do a lot in a day
	10	I think I do very little in a day
	17	I get little done
Reduced motivation	4	I feel like doing all sorts of nice things
	9	I dread having to do things
	15	I have a lot of plans
	18	I don’t feel like doing anything

In this study, fatigue was dichotomized into no reported fatigue (level one to two on the scale), and reported fatigue (level three to five on the scale) only on a sub-scale level when analysing the percentage distribution of participants rating fatigue. To simplify the dichotomization into no reported fatigue and reported fatigue some of the items scale (items 2, 5, 9, 10, 13, 14, 16, 17, 18 and 19) were converted to the reverse. In contrast, the total original score for fatigue was used in the correlation analysis and in the regression model.

#### Factors

Basic demographics of the participants were explored with *gender* and *age,* where age was divided into four following age groups: 18–29 years, 30–49 years, 50–69 years, equal and over 70 years. *Country of birth* the participants was divided in the two groups: persons born in Nordic countries (Sweden, Denmark, Finland and Norway), and persons born outside the Nordic countries (Afghanistan, Bolivia, Chile, Ethiopia, countries of the former Yugoslavia, Kosovo, Macedonia, the Philippines, Gambia, Iraq, Iran, Lebanon, Nigeria, Peru, Sierra Leone, Somalia, Syria, Tunisia, and Turkey). *Occupation/employment level* of the persons was explored by forming the two groups: working (working fulltime, working halftime or less), and not working ((unemployed, early retirement (pension before 65 years of age) and retired (pension at 65 years of age, or older)). *The use of mobility assistive devices* was divided into two following groups: not using mobility assistive devices and using mobility assistive devices (crutches, cane and/or walker, wheelchair occasionally, and wheelchair).

### Statistical analysis

Descriptive statistics were carried out on the participants’ characteristics and fatigue. Pearson’s correlation coefficient was used to analyse the strength in correlation between variables. A multiple linear regression was used to explore explanatory factors for fatigue. Prior to analyzing the multiple linear regression, the criteria of this method was reviewed for the nature of the underlying relationships and residuals. Data were analysed using the IBM SPSS Statistics, version 22.0 (IBM Corp., Armonk, NY, 2013).

## Results

### Characteristics of the participants

The mean age of the participants was 61 years (age range 19–93). Approximately half of the sample was female (54%), and the majority had PPS (99%). The occupation/employment level varied from unemployed (7%), working part time (6%), working full time (31%), early retirement (8%), and retired (48%). More than half of the participants used no mobility walking devices (66%), and among the participants who used mobility assistive devices, the use of assistive devices ranged from walking with crutches, cane and/or walker to using a wheelchair (Table 2).

**Table 2:** Characteristics of participants (*n* = 89)

Participants	*n* (%)
Gender	
Female	48 (54)
Age (years)	
Range	19–93 years
19–29	5 (6)
30–49	12 (13)
50–69	40 (45)
70 ≥	32 (36)
Country of birth	
Nordic countries	65 (73)
Outside the Nordic countries	24 (27)
Post-polio syndrome (PPS)	88 (99)
Occupation/employment level	
Unemployed	6 (7)
Working ≤50%	5 (6)
Working 100%	28 (31)
Early retirement	7 (8)
Retired	43 (48)
Use of assistive devices	
No walking devices	59 (66)
Crutches, cane and/or walker	20 (22)
Wheelchair occasionally	5 (6)
Wheelchair	5 (6)

### Fatigue

Overall, participants rated a high grade of fatigue in three of the five sub-scales of the MFI-20. The majority reported physical fatigue (90%), general fatigue (89%) and reduced activity (84%). Moreover, half of the sample reported mental fatigue and reduced motivation (Table 3).

**Table 3:** Percentage distribution and number of participants rating fatigue

MFI-20 Subscales	*n* (%)
General fatigue (4 items) (*n* = 89)	79 (89)
Physical fatigue (4 items) (*n* = 89)	80 (90)
Mental fatigue (4 items) (*n* = 88)	45 (51)
Reduced activity (4 items) (*n* = 87)	73 (84)
Reduced motivation (4 items) (*n* = 87)	45 (52)

### Correlation between fatigue and the factors

Fatigue positively and weakly correlated with age (*r* = 0.234, *p* < 0.05) and using mobility assistive devices (*r* = 0.255, *p* < 0.05), meaning that the older participants reported more fatigue than the younger and that the participants using mobility assistive devices reported less fatigue. The use of mobility assistive devices also correlated positively and weakly with occupational employment level (*r* = 0.238, *p* < 0.05) meaning that participants not working used mobility assistive devices to a lesser extent. Finally, there was a weak and positive correlation between country of birth and occupational/employment level (*r* = 0.278, *p* < 0.01) indicating that persons born in the Nordic countries were older and so thereby more often retired (Table 4).

**Table 4:** Correlation between fatigue and the factors (*n* = 85)

Variables	Fatigue	Gender	Age	Country of birth	Occupation/employment level
Fatigue	1				
Gender	−0.046	1			
Age	0.234*	−0.073	1		
Country of birth	−0.189	0.211	−0.729**	1	
Occupation/employment level	−0.017	−0.158	−0.548**	0.278*	1
Mobility assistive devices	0.255*	−0.004	−0.090	−0.74	0.238*

*p < 0.05, **p < 0.01

### Linear regression model

The multiple linear regression model showed that the factors age (β = 0.304, *p* < 0.019) and the use of mobility assistive devices (β = 0.262, *p* < 0.017) partially explained fatigue among persons living with late effects of polio. This result indicated that both high age and people not using mobility assistive devices reported more fatigue. There remaining two factors did not significantly explain fatigue in the final model. The regression model explained 14% of the variation in self-rated fatigue (Table 5).

**Table 5:** Multiple linear regression for fatigue and the factors (*n* =85)

Independent variables	β	95% CI	*p*-value
Age	0.304	0.38–4.04	0.019
Gender	−0.009	−2.70–2.28	0.932
Occupation/employment level	0.086	−2.20–4.36	0.514
Mobility assistive devices	0.262	0.63–6.22	0.017

*R* ^2^ = 14%

## Discussion

The aim of the present study was to explore possible factors associated with fatigue among persons with late effects of polio in Sweden. The main results were that persons not using mobility assistive devices and with high age reported more fatigue. However, the variation in fatigue could only be explained by 14% of the factors within the regression model.

In the present study, one of the factors that partly could explain the dependent variable of fatigue was the use of mobility assistive devices. A possible explanation for this could be that persons using mobility assistive devices stored their energy by simply using their mobility assistive devices, which in turn had a positive effect on the people’s self-rated fatigue. Also, persons not using mobility assistive devices might consume more energy when moving around, which may have resulted in an increased subjective feeling of fatigue. This finding of the importance of using mobility assistive devices is in line with a previous study [23], which showed that pain and fatigue could be associated with a lack of using mobility assistive devices among persons living with late effects of polio. On the other hand, another possible explanation could be that the persons with less fatigue did not need to use mobility assistive devices in a larger extent. Thus, the degree of fatigue did not lead to a need of using mobility assistive devices.

The findings from the present study indicated that age could contribute to explain fatigue among persons living with late effect of polio. This result may not only represent persons living with late effects of polio, it could also be transferred to people living with disability where fatigue is one of the symptoms. Moreover, gender and occupation/employment level could not significantly explain fatigue in the multiple regression model. This is in contrast to what has been shown in the general population, where females had higher mean values in all MFI-20 subscales compared to males [27]. One reason could be due to the small sample size. Concerning the occupation/employment level, the characteristics of the sample where more than half of the participants were not working (unemployed, early retirement, and retired) may explain this factor’s inability to explain the fatigue experienced.

Participation in many life areas might be restricted by increased fatigue. In the present study, mobility assistive devices have partly shown to have an association with fatigue, which implies that it is important to consider the access to mobility assistive devices in the healthcare system. Fatigue is a complex phenomenon [6], and therefore a number of factors that might be important for the understanding of fatigue should be included in the management of fatigue in this population. With this in mind, there is a need to consider the intersection of dimensions such as gender, age, cultural background, and occupational level.

In this retrospective registry study, fatigue was explored in relation to a set of factors that was taken from a clinical database. One limitation of using a clinical database was that it comprised predetermined variables, which restricted the choice of other factors of importance for the present study. Earlier qualitative studies [21, 22] have shown that life conditions related to conditions for daily occupations, social support, social network and municipal service were important for immigrants living with late effects of polio. These aspects could not be captured in the present study due to the use of predetermined variables.

Fatigue was assessed with the MFI-20 [5]. There are several questionnaires to measure fatigue [6, 25, 28, 29], but fatigue is a widely experienced symptom [5], that might vary rapidly [30]. The MFI-20 is valid and reliable for measuring fatigue in persons with PPS [25], and was therefore a suitable questionnaire to capture fatigue. Furthermore, a limited number of factors were explored in relation to fatigue in the present study due to the small sample size which is a limitation. Therefore, further research is needed to explore other relevant explanatory factors.

## Conclusion

Persons not using mobility assistive devices and high age reported more fatigue. Therefore, fatigue could partly be explained by the extent of using mobility assistive devices and the people’s age. These two factors are important, but to understand the complex nature of fatigue, additional factors must be considered. Healthcare professionals should provide and demonstrate the importance of assistive devices to ensure management of fatigue in persons living with late effects of polio. This knowledge contributes with an additional piece in the puzzle to better understand fatigue among persons living with late effects of polio.

## Abbreviations

EMG: Electromyogram
GF: General fatigue
MF: Mental fatigue
MFI: Multidimensional fatigue inventory
PF: Physical fatigue
PPS: Post-polio syndrome
RA: Reduced activity
RM: Reduced motivation
